# A trans-umbilical single-site plus one robotic-assisted surgery for choledochal cyst resection in children

**DOI:** 10.3389/fped.2024.1418991

**Published:** 2024-06-18

**Authors:** Yucan Lin, Shan Chen, Yang Lin, Ling Zhang, Jianbin Wang, Xinyi Qiu, Di Xu, Lizhi Li

**Affiliations:** ^1^ Department of Pediatric Surgery, Fujian Provincial Hospital, Fujian Provincial Clinical Medical College of Fujian Medical University, Fuzhou, Fujian, China; ^2^ Department of Laboratory, Fuzhou Second General Hospital, Fuzhou, Fujian, China

**Keywords:** trans-umbilical single-site plus one, robotic-assisted, laparoscopy, choledochal cyst, children

## Abstract

**Objective:**

The purpose of this study is to compare the intraoperative and postoperative outcomes of a trans-umbilical single-site plus one robot-assisted surgery and a trans-umbilical single-site laparoscopic surgery in the treatment of choledochal cysts.

**Methods:**

We retrospectively analyzed clinical data from 49 children diagnosed with choledochal cysts who were admitted to our hospital between June 2020 and December 2023. Among these patients, 24 underwent a trans-umbilical single-site plus one Da Vinci robot-assisted surgery (the robot group) and 25 underwent a trans-umbilical single-site laparoscopic-assisted surgery (the laparoscopic group). We compared differences in intraoperative and postoperative outcomes between the two groups.

**Results:**

There was no significant difference between the two groups of patients in terms of gender, age, weight, clinical symptoms, maximum cyst diameter, type, postoperative complications, and facial expression, leg movement, activity, crying, and comfortability (FLACC) scoring (*p* > 0.05). Compared with the patients in the laparoscopic group, those in the robot group had less intraoperative bleeding [10 (8–12) vs. 15 (11.5–18) ml, *p *< 0.001] and required less postoperative drainage tube indwelling time [5 (4–6) vs. 7 (5.5–8) day, *p *< 0.001], less postoperative fasting time [4 (3–4) vs. 6 (5–7) days, *p *< 0.001], and less postoperative hospitalization time [6 (6–7) vs. 8 (6–10) days, *p *< 0.001], but they required more operative time [385.5 (317.0–413.3) vs. 346.0 (287.0–376.5) min, *p *= 0.050] and consumed more hospitalization expenses (79,323 ± 3,124 vs. 31,121 ± 2,918 yuan, *p *< 0.001).

**Conclusion:**

The results of this study showed a shorter hospitalization time, quicker postoperative recovery, and less tissue damage but a higher cost and a longer operation time in patients who chose robotic surgery rather than laparoscopic surgery. With the continuous expansion of the scale of installed robot-assisted surgical systems and the gradual accumulation of the technical experience of surgeons, robot-assisted surgery may slowly surpass, and shows a trend to replace, laparoscopy because of its advantages.

## 1 Introduction

Choledochal cysts (CCs) are commonly congenital biliary malformations including both biliary local dilatation and pancreaticobiliary maljunction (1), which have an incidence rate that is higher in Asia than in Europe and America (2). The fundamental treatment of choledochal cysts is surgery (3), and it is one of the more complex procedures in pediatric hepatobiliary surgery. In the past 10 years, with the application and popularization of minimally invasive techniques in pediatric surgery, laparoscopic surgery for choledochal cysts has gradually replaced traditional laparotomy and become the gold standard for the treatment of choledochal cysts (4). The Da Vinci robotic-assisted technique represented great progress in the field of minimally invasive surgery. Since the first reported robotic-assisted resection of choledochal cysts in children in 2006, and after Huang et al. (5) completed the first robotic-assisted surgery for choledochal cyst resection in China in 2009, this operation was reportedly performed successively at home and abroad. At present, robotic-assisted laparoscopic radical resection for choledochal cysts is still in the exploratory stage in China, and the data comparing the efficacy of a trans-umbilical single-site plus one robotic-assisted and a single-port laparoscopic surgery for choledochal cysts are limited. Therefore, the purpose of this study was to compare the efficacy of the two surgeries for choledochal cysts.

## 2 Materials and methods

### 2.1 Clinical information

We retrospectively analyzed the clinical data of 49 children with congenital choledochal cysts admitted to Fujian Provincial Hospital between June 2020 and December 2023. The design of the study was approved by the Ethics Committee of Fujian Provincial Hospital (No. 2020-KY-018). All patients underwent ultrasonography and magnetic resonance cholangiopancreatography (MRCP) after birth to confirm the diagnoses of CCs and Todani types (6). Among them, 24 patients underwent a trans-umbilical single-site plus one robotic-assisted surgery for radical choledochal cyst resection (the robot group), and the remaining 25 patients underwent single-site laparoscopic treatment (the laparoscopic group). The parents of the patients chose the surgical strategy based on their preferences, with the preoperative general condition of the two groups of children being deemed fit to proceed with the two operating methods. The surgical procedures were carried out by staff with adequate experience.

The inclusion criteria were as follows: (1) the presence of congenital choledochal cysts diagnosed by scanning medical records and imaging examination results and treated by a surgical procedure; (2) the absence of any digestive system malformation; (3) patients with no previous history of abdominal surgery; (4) those with no abnormality of coagulation function and no other important organ dysfunction (5) patients who had no missing clinical data and underwent postoperative follow-up.

The exclusion criteria were as follows: (1) patients with Caroli syndrome; (2) perforation or malignant transformation of choledochal cysts; (3) those with a history of abdominal surgery or repeated severe infection of the biliary system; and (4) those with incomplete clinical data or those who did not consent for follow-up after the operation.

### 2.2 Surgical techniques

All operations were performed by the same surgical team.

#### 2.2.1 Trans-umbilical single-site plus one robotic-assisted resection surgery

**Figure 1:**
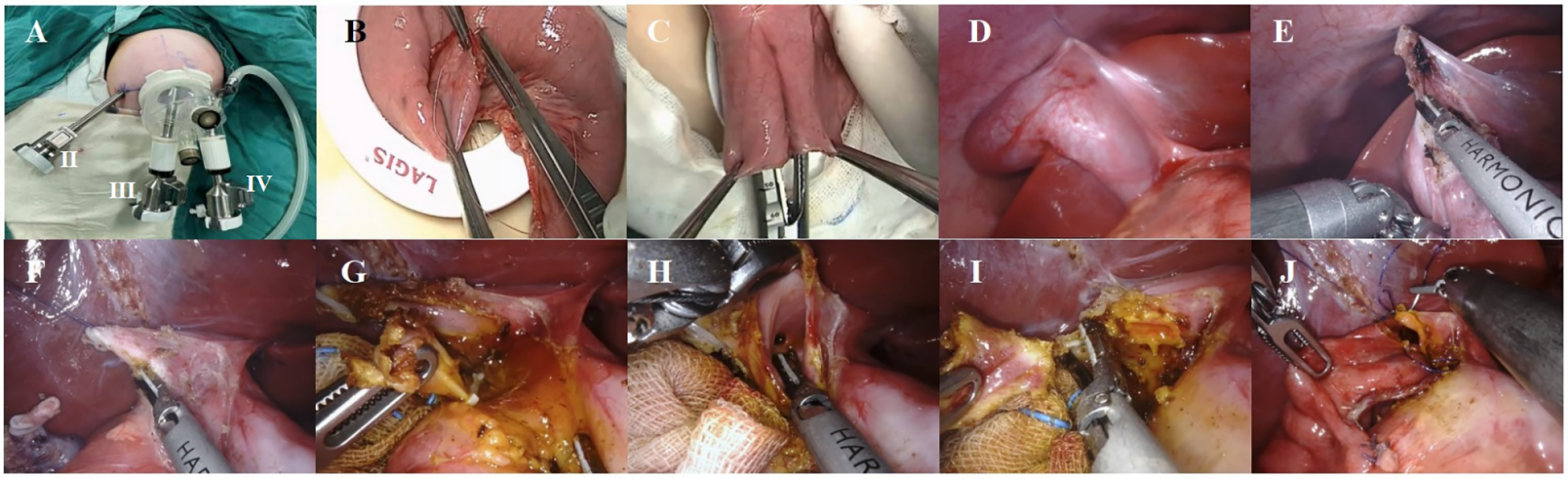
(**A**) Location of robotic trocars; (**B**) the baseplate of the device can serve as a container; (**C**) extracorporeal Roux-en-Y jejunojejunostomy; (**D**) suspension of the gallbladder fundus; (**E**) removing the gallbladder with an ultrasonic scalpel; (**F**) cutting the cyst's anterior wall and decompressing the cyst; (**G**) ligating the distal opening of the choledochal cyst with Hem-o-lok polymeric clips; (**H**) detection of the common hepatic duct; (**I**) cyst and trimming the hepatic duct of the hilar part; (**J**) end-to-side hepaticojejunostomy.

Following the administration of general anesthesia and endotracheal intubation, each patient was positioned in a supine posture with their head elevated 30° and tilted 30°–45° to the left, while also being fitted with a catheter and stomach tube. An arc incision was made around the umbilicus and a quadruple-channel puncture device was placed to establish a single-site channel (Figure 1A). The device was then removed and the baseplate kept, and the jejunum was grasped with intestinal forceps and lifted out of the body through the baseplate to perform an extracorporeal Roux-en-Y jejunojejunostomy. The jejunum was severed 25 cm away from the Treitz ligament with a linear cutting stapler (Figure 1B), followed by a side-to-side jejunojejunostomy with the distal jejunum 20 cm from the transection point (Figure 1C). The distal jejunal loop was pulled to the hepatic hilum through the posterior transverse colon tunnel. The puncture device was reset, and the pneumoperitoneum was re-established. The puncture device had four channels; two larger 1.2 cm channels were placed into an 8 mm 3D camera port III and an 8 mm operating port IV, and the other two 5 mm operation channels were used as assistant ports. Another 8 mm operation sheath of robotic operating port II was made and was placed 6 cm to the right of the umbilicus, and the distance between the operation sheaths of port II, port III, and port IV was approximately 4 cm. The gallbladder fundus and hepatic round ligament were pulled to the abdominal wall (Figure 1D) and the gallbladder and common bile duct were disassociated to expose the cyst (Figure 1E). The anterior cyst wall was cut, the bile sucked, and the posterior cyst wall (Figure 1F) separated to the confluence of the distal end of the cyst and pancreatic duct. The distal end of the common bile duct was then clamped with Hem-o-lok polymeric clips (Figure 1G), the distal cyst wall resected, the gallbladder and common bile duct cyst excised together, the opening or non-opening of the left and right hepatic ducts confirmed (Figure 1H), and the common liver duct trimmed to be trumpet-shaped (Figure 1I). An absorbable suture was used for end-to-side hepaticojejunostomy. The distal jejunal loop and the common hepatic duct were matched, the medial angle of the incision was sutured, and the posterior wall and anterior wall of the common hepatic duct–intestinal duct were sutured continuously in turn (Figure 1J). A washing of the sutured area showed that there was no biliary fistula and bleeding and that the incision was sutured properly (7).

#### 2.2.2 Trans-umbilical single-site laparoscopic-assisted resection procedure

After the application of general anesthesia and endotracheal intubation, a quadruple-channel puncture device was inserted through an arc incision around the umbilicus, the gallbladder and hepatic round ligament were suspended in turn, and the cyst of the gallbladder and common bile duct was removed. The quadruple-channel puncture device was removed but the baseplate was retained, and the jejunum was grasped with intestinal forceps and lifted out of the body through the baseplate to perform an extracorporeal Roux-en-Y jejunojejunostomy. The intestine was returned to the abdominal cavity. The distal jejunum loop was passed under the transverse mesocolon and endoscopic hepaticojejunostomy was completed.

### 2.3 Intraoperative and postoperative data collection

We observed and recorded the intraoperative and postoperative conditions of the two patient groups in terms of operation time, intraoperative bleeding, and postoperative hospitalization time (the discharge standards were stable vital signs, close to normal value clinical examination, restored normal diet and stable defecation function, and no complications such as incision infection and biliary leakage). Three days after the operation, postoperative pain was measured using the modified facial expression scoring method (FLACC scoring including facial expression, leg movement, activity, crying, and comfortability. For each behavior, 0–2 points are awarded; the sum of the five indexes is 0 points at the lowest and 10 points at the highest. The higher the score, the more obvious the discomfort and pain). Postoperative complications (including biliary or pancreatic leakage, anastomotic stenosis, pancreatitis, cholangitis, intestinal obstruction, infection, etc.), postoperative drainage tube indwelling time (the tube would not be removed until the abdominal drainage fluid was clear, its volume was below 10 ml, and there were no complications such as biliary and pancreatic leakage), and postoperative fasting time (the patients were fasted until the occurrence of bowel activity; water was given first, followed by a liquid and a then a soft diet.) were studied. Finally, the total hospitalization expenses were calculated in detail.

### 2.4 Statistical analysis

IBM SPSS Statistics 26 was used to analyze the data. Mean ± standard deviation (SD) was used to express the measurement data following normal distribution, and an independent sample *t*-test was used to examine these data. Median and interquartile range (IQR) was used for those whose measurement data did not conform to normal distribution, and the Mann–Whitney *U* test was used to examine these data. The enumeration data were expressed as the number of cases (%), and a comparison between the two groups was made using Pearson's χ^2^ and Fisher's exact tests. *p*-values < 0.05 were considered statistically significant.

## 3 Results

The demographic characteristics of patients who were diagnosed with choledochal cysts and underwent surgical treatment are summarized in Table 1. In the robot group, there were 8 boys (33.3%) and 16 girls (66.7%), with an age median of 26 months (6–146 months) and a weight median of 13 kg (range, 6.9–40.9 kg). Of these patients, 14 (58.3%) remained asymptomatic and only the color Doppler ultrasound showed choledochal cysts; 10 (41.7%) developed symptoms before surgery. Percentages according to the Todani type were 75.0 in Ⅰ, 4.2 in Ⅱ, 20.8 in Ⅲ, and 0 in IV. The maximum diameter of the choledochal cysts in MRCP was 3.40 ± 1.33 cm. In the laparoscopic group, there were 10 boys (40.0%) and 15 girls (60.0%), with an age median of 26 months (9–132 months), and a weight median of 14.6 kg (range, 9.2–57.6 kg). Among them, 13 (52.0%) remained asymptomatic and only the color Doppler ultrasound showed choledochal cysts; 12 (48.0%) developed symptoms before surgery. Percentages according to the Todani type were 72.0 in Ⅰ, 4.0 in Ⅱ, 4.0 in Ⅲ, and 20.0 in 2163;. In this group, the maximum diameter of the choledochal cysts in MRCP was 3.16 ± 1.06 cm. There was no significant difference between the two groups in terms of gender, age, weight, clinical symptoms, maximum cyst diameter, and type (*p* > 0.05).

**Table 1:** Comparison of the demographic characteristics of the two surgical groups.

Group	Sample number	Sex (%)		Age (months), M (IQR)	Weight (kg), M (IQR)	Symptom (%)		The maximum cyst diameter (cm), mean ± SD	Todani type (%)	
		Male	Female			Yes	No		Type I	Other types (Ⅱ, Ⅲ, and Ⅳ)
RS	24	8 (33.3)	16 (66.7)	26 (11.5–45)	13 (10.63–16.3)	10 (41.7)	14 (58.3)	3.40 ± 1.33	18 (75.0)	6 (25.0)
LS	25	10 (40)	15 (60)	24 (18.5–38.5)	14.6 (12.4–17.3)	12 (48)	13 (52)	3.16 ± 1.06	18 (72.0)	7 (28.0)
*t* (Z, *χ*²)	—	0.234		−0.280	−1.501	0.199		0.690	0.057	
*p*	—	0.628		0.779	0.133	0.656		0.494	0.812	

RS, robotic surgery; LS, laparoscopic surgery; M, median; IQR, interquartile range; mean ± SD, mean ± standard deviation.

After completing the operation in both groups of children, no conversion to open surgery was made. Intraoperative bleeding, postoperative drainage tube indwelling time, postoperative fasting time, and postoperative hospitalization time in the robot group were significantly less than those in the laparoscopic group (*p *< 0.05), while the operation time and the hospitalization expenses in the robot group were significantly greater than those in the laparoscopic group (*p *< 0.05), as seen in Table 2. In the laparoscopic group, one child developed ascites on the fourth day after the operation, which was cured by ultrasound-guided puncture and drainage. In the robot group, one child developed an abdominal infection on the second day after the operation, which was cured after anti-infection treatment with cefoperazone. In the laparoscopic group, two children had abdominal infections on the third day after the operation, which were cured after cefoperazone anti-infection treatment. One patient developed an incision infection on the fifth day after the operation, which was cured by debridement, dressing change, and anti-infection measures. There was no significant difference in postoperative complications and FLACC scoring between the two groups (*p* > 0.05). All patients were told to return to the hospital for a re-examination of abdominal color Doppler ultrasound and liver function at 1, 3, and 6 months after discharge, and an abdominal CT examination was performed if necessary to determine whether there were complications. The median follow-up time of the robot and the laparoscopic groups were 18 and 20 months, respectively. Up to now, no long-term complications have been found in the children at follow-up.

**Table 2:** Comparison of intraoperative and postoperative conditions of the two surgical groups.

Group	Operative time^a^ (min), M (IQR)	Intraoperative bleeding (ml), M (IQR)	Complications (%)		Postoperative drainage tube indwelling time (days), M (IQR)	FLACC scores, mean ± SD	Postoperative fasting time (days), M (IQR)	Postoperative hospitalization time (days), M (IQR)	Hospitalization Expenses (RMB), mean ± SD
			Yes	No					
RS	385.5 (317.0–413.3)	10 (8–12)	1 (4.2)	23 (95.8)	5 (4–6)	1.05 ± 0.30	4 (3–4)	6 (6–7)	79,323 ± 3,124
LS	346.0 (287.0–376.5)	15 (11.5–18)	4 (16.0)	21 (84.0)	7 (5.5–8)	1.13 ± 0.29	6 (5–7)	8 (6–10)	31,121 ± 2,918
*t* (Z, χ²)	−1.960	−4.355	1.871		−3.685	−1.006	−4.855	−3.619	57.486
*p*	0.050	<0.001	0.187		<0.001	0.320	<0.001	<0.001	<0.001

RS, robotic surgery; LS, laparoscopic surgery; M, median; IQR, interquartile range; mean ± SD, Mean ± standard deviation.

^a^ Operation time was the period from incising the skin at the initial to the last stage of wound closure at the end of the operation.

## 4 Discussion

The main symptoms of choledochal cysts are abdominal pain, vomiting, jaundice, and abdominal mass (8). If these are not effectively treated, children with choledochal cysts may experience perforation, cholangitis, liver failure, and eventually bile duct tumors. Surgical resection of choledochal cysts appears to be the main treatment for choledochal cysts at present; Roux-en-Y hepaticojejunostomy and extrahepatic bile duct tree resection have been performed as open procedures (9). Minimally invasive surgery for treating choledochal cysts opened a new chapter in the laparoscopic treatment of choledochal cysts after the report of Farello et al. in 1995 (10). At present, there are a large number of case reports about this technique both at home and abroad, which proves that it is safe and effective in treating choledochal cysts (11). At the same time, robotic-assisted surgery, has been gradually applied to the treatment of choledochal cysts. However, with a relatively narrow abdominal space, limited operating space, and expensive surgical supporting equipment, robotic-assisted surgery has made slow progress in the area of pediatric surgery and is still in the exploratory stage because of the requirement for multiple large-aperture Trocar incisions. In 2006, Woo et al. (12) reported the first robotic-assisted surgery in children with choledochal cysts and achieved successful results; this has been suggested as a new development direction for the alternative treatment of choledochal cysts (13).

Because of less traumatic incidents, strong recovery ability, and a clear and deep anatomical field of vision, both laparoscopic and robot-assisted surgery were considered superior to traditional laparotomy in intraoperative and postoperative prognoses (14). However, to date, laparoscopic choledochal cyst resection remains a highly challenging procedure (15), mainly because of the long learning curve and the technical complexities involved, especially for a laparoscopic anastomosis of the common hepatic ducts and jejunum, which requires accurate suturing and a narrow operation space and increase the operational difficulty of needle holders and limit the procedure’s clinical application. In addition, endoscopic instruments can only be used back and forth, and the accuracy and safety of choledochojejunostomy will be affected by factors such as expansion and rotation, poor tactile feedback, the fulcrum effect of instruments on the abdominal wall, a lack of three-dimensional imaging, and poor ergonomics. However, robot-assisted surgery can effectively make up for the shortcomings of laparoscopic surgery, and its advantages in treating choledochal cysts are given in the following paragraphs.

First, the information system has three-dimensional visualization, which makes the visual field mirror clearer and more comprehensive. A robotic-assisted surgical system provides a brand-new, high-resolution, high-precision, and high-definition view for surgery and can automatically enlarge the visual field by 10–15 times. At the same time, it provides a shaking and filtering function, which makes the image more stable and accurate, enables a more detailed operation under a clear visual field, avoids damage to important organs such as the right hepatic artery and portal vein, and separates more carefully, accurately peeling off bile duct cysts to the pancreatic section and greatly reducing wound exudation. Therefore, robot-assisted surgery is particularly important for microtubular structures such as bile ducts. In the robotic group in this study, there were three patients with severe adhesion around the cyst, and the robot-assisted surgical system could accurately separate the cysts and completely remove them, thereby avoiding residual cysts; such a process will not cause damage to the bile ducts and pancreatic ducts and will facilitate the exploration of distal bile ducts.

Second, the robotic system has a flexible arm, can carry out a multiangle precise rotation, imitate the movement, bending, closing, and rotation of human hands, carry out precise grasping, dissociating, cutting, and suturing, and can reach 7 degrees of freedom, thus overcoming the shortcomings of stiff and inflexible common endoscopic instruments. It can carry out motion scaling, reduce the action ratio of the operator in the range of 5:1, thus carrying out fine separation and suturing in a small space, improve flexibility and range of motion, increase the success rate of choledochojejunostomy, and reduce the incidence of postoperative bile leakage and the hospitalization days of patients. Accurate suturing with the help of the robot greatly accelerates the recovery speed of children and reduces hospitalization time. In this study, the children in the robotic group had less bleeding and no bile leakage after the operation. In addition to the sterile area, the operator can work comfortably on the super console, and its ergonomic design can also reduce the physical exertion of the operator. The operation method of the robot-assisted surgery is similar to that of the traditional laparoscope surgery (16). Experience gained in handling a laparoscope helps shorten the learning curve of the robotic procedure and aids in mastering it better than the laparoscope. With the improvement of operational techniques and the addition of trained auxiliary personnel and equipment nurses, the operation time of robots will become shorter.

In this study, the operation time of the robotic procedures of the first three children was approximately 7 h, which can be rapidly shortened to 4 h with the improvement of surgical skills. In a narrow space, robotic surgery provided huge operational scope, and has the experience of successful anastomosing 2 mm accessory hepatic ducts (17). In recent years, many studies at home and abroad have shown that, compared with traditional laparoscopy, robotic surgery has unique advantages in terms of reduced postoperative anastomotic complications (such as biliary fistula and anastomotic stenosis) (18), but there is still a lack of large-sample research. In this context, by comparing the operations performed by the same group of doctors in the same period, we found that the amount of bleeding, the time of indwelling abdominal drainage tube [10 (8–12) vs. 15 (11.5–18) days, *p *< 0.05], the fasting time [4 (3–4) vs. 6 (5–7) days, *p *< 0.05], and the hospitalization time [6 (5–7) vs. 8 (6–10) days, *p *< 0.05] were all significantly lower than those of laparoscopy and inseparable from the advantages of robotic surgery. Compared with traditional laparoscopy, a robotic surgical system has higher precision, a finer anatomical structure, and a more comprehensive visual field, causes less damage to the blood vessels and important tissues and organs and less postoperative inflammatory reaction, releases less exudates such as bile, and promotes tissue repair and the recovery of gastrointestinal function, which is also the main reason for reduced abdominal drainage time, fasting time, and discharge time in robotic surgery. In addition, the operation time and average hospitalization expenses were higher in the robotic treatment group of patients than in the endoscopic surgical group of patients in this study, and there were also significant differences between them (both *p *< 0.05). Among them, one patient had an abdominal infection and three had cholangitis and biliary fistula, but there was no statistical significance, which may be due to the small number of patient cases and short follow-up time and so requires further study.

Due to the technical limitations of developing instruments suitable for children, many recent studies have reported related improvement methods such as reducing operation sheaths and increasing auxiliary holes, but there are some shortcomings such as unsatisfactory appearance, poor scar concealment, and unconcentrated surgical incision position. In this study, continuous traction was used instead of traction during the operation, and the ligament around the liver, the gallbladder bed at the bottom of the gallbladder, common bile ducts, and common liver ducts were pulled and fixed in turn during the operation, which not only kept the ideal appearance of the incision in the single-port laparoscopic operation, but also increased the scope of the operation and reduced the difficulty of its performance. Among the children included in the study, the youngest was 6 months old and their weight was 6.9 kg. Jin et al. (19) reported that 10 children under 6 kg successfully received a robot-assisted laparoscopic radical choledochal cyst surgery. It is hoped that with the continuous improvement of surgical techniques, the adaptation range of pediatric surgery will become increasingly wider.

However, robot-assisted choledochal cyst surgery also has the following shortcomings: First, the average hospitalization cost of the robotic group is 48,202 yuan higher than that of the laparoscopic group (79,323 ± 3,124 vs. 31,121 ± 2,918 yuan, *p *< 0.001). Second, it is necessary to set up a special robotic operating room with professionally trained assistants and instrument nurses. Third, it takes a long time for the robotic arm to locate, and the machine must be recalibrated and adjusted when changing the body position during the operation, which will prolong the operation time. Fourth, robotic surgical equipment is huge in size, the selection of instruments is relatively limited, and the size of the equipment port position makes its use in children limited. Fifth, a lack of tactile feedback may cause damage to surrounding tissues, fracture of sutures, and poor control of suture tightness. Furthermore, trocar is too large, which limits its application in pediatric surgery, especially in neonatal surgery.

## 5 Conclusion

The results of this study showed a shorter hospitalization time, quicker postoperative recovery, and less tissue damage but a higher cost and longer operation time in patients who chose robotic surgery rather than laparoscopic surgery. With the continuous expansion of the scale of installed robot-assisted surgical systems and the gradual accumulation of technical experience by surgeons, robot-assisted surgery may slowly surpass, and shows a trend to replace, laparoscopy because of its advantages.

## Data Availability

The original contributions presented in the study are included in the article/Supplementary Material, further inquiries can be directed to the corresponding authors.
